# Reduced Autophagy by a microRNA-mediated Signaling Cascade in Diabetes-induced Renal Glomerular Hypertrophy

**DOI:** 10.1038/s41598-018-25295-x

**Published:** 2018-05-03

**Authors:** Supriya Deshpande, Maryam Abdollahi, Mei Wang, Linda Lanting, Mitsuo Kato, Rama Natarajan

**Affiliations:** Department of Diabetes Complications and Metabolism, Diabetes Metabolism Research Institute and Beckman Research Institute of City of Hope, Duarte, California 91010 USA

## Abstract

Autophagy plays a key role in the pathogenesis of kidney diseases, however its role in diabetic nephropathy (DN), and particularly in kidney glomerular mesangial cells (MCs) is not very clear. Transforming Growth Factor- β1 (TGF-β), a key player in the pathogenesis of DN, regulates expression of various microRNAs (miRNAs), some of which are known to regulate the expression of autophagy genes. Here we demonstrate that miR-192, induced by TGF-β signaling, plays an important role in regulating autophagy in DN. The expression of key autophagy genes was decreased in kidneys of streptozotocin-injected type-1 and type-2 *(db/db)* diabetic mice and this was reversed by treatment with Locked Nucleic Acid (LNA) modified miR-192 inhibitors. Changes in autophagy gene expression were also attenuated in kidneys of diabetic *miR-192*-KO mice. *In vitro* studies using mouse glomerular mesangial cells (MMCs) also showed a decrease in autophagy gene expression with TGF-β treatment. miR-192 mimic oligonucleotides also decreased the expression of certain autophagy genes. These results demonstrate that TGF-β and miR-192 decrease autophagy in MMCs under diabetic conditions and this can be reversed by inhibition or deletion of miR-192, further supporting miR-192 as a useful therapeutic target for DN.

## Introduction

The burden of diabetes and associated complications is increasing worldwide indicating an urgent need to identify new therapeutic targets. Diabetic Nephropathy (DN) is a common microvascular complication of diabetes and a major cause of end stage renal disease (ESRD). The main features of DN include proteinuria, albuminuria, renal glomerular hypertrophy, basement membrane thickening, podocyte dysfunction, mesangial and tubulointerstitial fibrosis due to accumulation of extracellular matrix (ECM) proteins^1,2^. Transforming growth factor-ß1 (TGF-ß) is one of the most important mediators of fibrosis as well as cellular hypertrophy and survival^1–4^. Expression of TGF-ß is increased in renal cells including mesangial cells (MC) and tubular cells in DN and plays a major role in the pathogenesis of DN^5,6^.

Autophagy is an intracellular catabolic process which maintains cellular homeostasis. It is a process by which long lived protein aggregates and damaged organelles are degraded via the formation of double membraned vesicles called autophagosomes^7–11^. Autophagic activity is enhanced under conditions of cellular stress such as nutrient starvation, oxidative stress and endoplasmic reticulum (ER) stress^7–14^. The different phases of autophagy include initiation of vesicle formation, vesicle elongation, fusion of vesicles with lysosomes and degradation of cytoplasmic contents. Each of these stages is regulated by a number of autophagy proteins. The Ulk1 (Atg1) Ser/Thr protein kinase complex initiates the process of autophagy. Beclin-1 is a subunit of the class III phosphoinositide-3-kinase (PI3K) complex which is important for triggering the formation of autophagosomes. The Atg5-Atg12 ubiquitin-like conjugation complex stimulates the elongation step and is also involved in the localization of Microtubule-associated protein 1A/1B-light chain 3 (LC3) to the autophagosomes. The soluble form of LC3 (LC3-I) is then converted to the autophagosomal membrane associated form LC3-II by conjugation of phosphatidylethanolamine (PE). Lipidated LC3-II remains associated with these vesicles and after the formation of autophagosomes is complete, these vesicles merge with lysosomes. This step is followed by sequestration of cytoplasmic contents for degradation. Sequestome 1 (SQSTM1/p62) is a protein that is distributed not only in the cytoplasm, but also localizes to the nucleus as well as to autophagosomes, and is preferentially degraded within these vesicles^15^. Accumulation of p62 in cells is indicative of a defect in the autophagic pathway^7–9,11,16,17^.

Studies from multiple groups have shown that autophagy plays a renoprotective as well as a pathogenic role in different kidney diseases^18–21^. Epidermal growth factor receptor Inhibition slows progression of DN in association with an increase in autophagy^22^. A very-low-protein diet ameliorates advanced DN through autophagy induction by suppression of the mTORC1 pathway in Wistar fatty rats, type 2 diabetes and obesity^23^. These reports suggest that restoring autophagy may prevent DN in animal models. Induction of autophagy genes and formation of autophagic vesicles has been demonstrated in renal ischemia-reperfusion animal models^11,12,18,19^. Renal cell culture studies have shown that autophagy leads to cell death during cellular stress^9,24^. On the contrary, studies have shown that there is an increased susceptibility to glomerular disease following loss of Atg5. Knockout of Atg5 in renal proximal tubular cells led to accumulation of misfolded proteins and organelles and increased susceptibility to ischemia-reperfusion injury^25^. Deletion of Atg5 in podocytes led to glomerulopathy as well as ER stress and proteinuria in aging mice^26^. Similarly, a protective role for autophagy has been demonstrated in cisplatin-induced renal injury mouse models as well as in renal cells^27,28^. A number of studies have shown that renal autophagy is impaired under diabetic conditions (in both type 1 and type 2 diabetes models) which leads to pathogenesis of DN^29,30^. Various signaling pathways such as mTOR, AMPK, Sirt1, as well as oxidative stress, hypoxia and ER stress which are altered under diabetic conditions have been implicated in the regulation of autophagy^20^. It is known that TGF-ß activates as well as inhibits autophagy in renal tubular cells, fibroblasts and also in certain cancer cells^29,30^. While the role of autophagy in renal tubular cells and podocytes has been well documented^29,30^, its role in MC has not been widely studied.

MicroRNAs (miRNAs), are short sequences of RNA which regulate gene expression and cellular processes^31–34^, TGF-ß regulates the expression of various miRNAs which have been well studied and implicated as regulators of renal dysfunction in various kidney complications including DN^35,36^. We have previously shown that miR-192 can mediate fibrotic effects of TGF-ß and plays a key role in the pathogenesis of DN in MC^37,38^. miR-192 is also part of a cascade of downstream miRNAs (including miR-200, miR-216a, miR-217) that also regulate fibrotic genes and contribute to the pathogenesis of DN^39,40^. A number of miRNAs have also been shown to regulate autophagy genes at different steps of the autophagic pathway in various cancer cells^41^. However, the role of miRNAs in the regulation of autophagy in MC under diabetic conditions remains unknown. In this study we demonstrate for the first time that TGF-ß and miR-192 regulate the expression of key genes associated with autophagy in MC *in vitro* as well as in renal glomeruli *in vivo* under diabetic conditions.

## Results

### Decreased expression of key autophagy genes in kidneys of diabetic *db/db* mice relative to control *db/*+ mice

**Figure 1:**
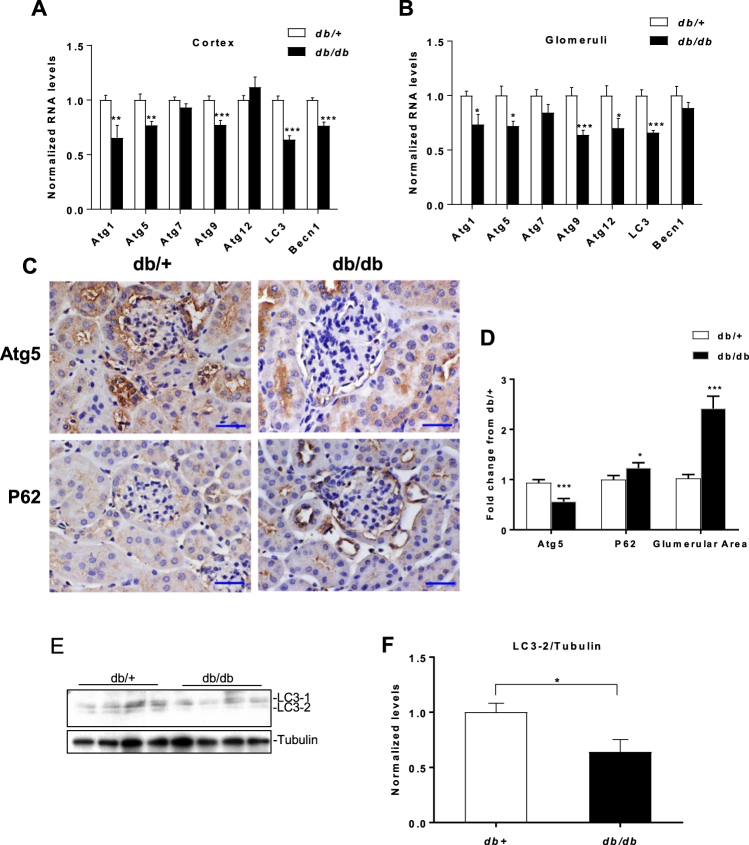
Decreased autophagy gene expression in kidneys of db/db mice relative to control *db/*+ mice.qRT-PCR analysis of autophagy genes in (**A**) kidney cortex lysates (n = 4 per group) and (**B**) glomerular lysates, from 10–12 week old - *db/db* mice and *db/*+ *mice* (n = 3 per group). (**C**) Representative immunostaining and (**D**) quantification for p62, Atg5 and glomerular area from cortical sections of *db/*+ and *db/db* mice; (n = 3 per group). Scale bar, 20 µm. x400 (10 × 40) magnification. (**E**) Western blot and (**F**) quantification of LC3 levels (LC3-2/Tubulin) from renal glomerular lysates of 10–12-week-old *db/*+ and *db/db* mice; (n = 4 per group). Uncropped scans are presented in Supplementary Fig. 1. *P < 0.05; **P < 0.01; ***P < 0.001.

In order to examine whether renal autophagy was impaired under diabetic conditions, we examined expression of autophagy genes in renal cortical lysates of 10–12 week old *Lepr*^*db/db*^ mice (type 2 diabetes model) (*db/db*). Expression of several genes (*Atg1*, *5*, *9*, *LC3 and Becn1*) was significantly decreased in cortical lysates of *db/db* mice compared with control *db/*+ mice, but not *Atg7* or 12 (Fig. 1A). A similar decrease in the expression of these genes was also observed in glomerular lysates from *db/db* mice compared with control *db/*+ mice (Fig. 1B). Next we analyzed protein expression of p62, an important marker of autophagy. Autophagy causes selective degradation of p62/SQSTM1 and a defect in this process leads to accumulation of p62 in cells^15^. Immunostaining for p62 in kidney cortex sections from *db/*+ and *db/db* mice showed increased levels of p62 in glomeruli of *db/db* mice compared to *db/*+ mice indicating a defect in autophagy under diabetic conditions (Fig. 1C,D). Next we performed immunostaining in renal sections for Atg5, an important regulator of autophagy, which showed that protein levels of Atg5 were decreased under diabetic conditions (*db/db* mice) compared to control (*db/*+ mice) (Fig. 1C,D). Glomerular area was increased in *db/db* mice compared to control *db/*+ mice as expected (Fig. 1C,D). Conversion of the cytosolic isoform of LC3-1, via an ubiquitination like reaction, to LC3-2 is a marker of autophagosomes in cells. Western blot analysis using glomerular lysates from *db/db* and control *db/*+ mice showed that conversion of LC3-1 to LC3-2 was significantly decreased in glomerular lysates from *db/db* mice compared to control *db/*+ mice (Fig. 1E,F). These results show that expression of several autophagy genes and proteins is decreased in cortical and glomerular lysates from type 2 diabetic mice compared to control mice, indicating an impairment of autophagy in this model of type 2 diabetes and DN.

### Decreased autophagy gene expression in kidneys of STZ injected diabetic mice and selective reversal of this effect with LNA-anti-miR-192

**Figure 2:**
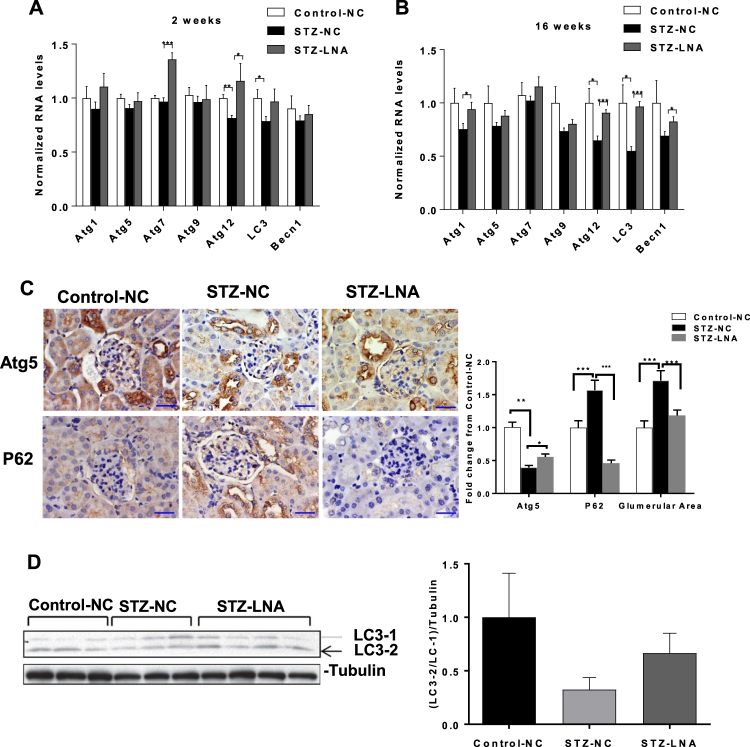
Decreased expression of autophagy genes in kidneys of STZ-injected diabetic mice relative to control mice and reversal of this effect on some genes with LNA-anti-miR-192 oligonucleotides.qRT-PCR analysis of autophagy genes in kidney cortex lysates from control (n = 3) and STZ-injected mice. (**A**) 2 weeks and (**B**) 16 weeks after induction of diabetes treated with either negative control (NC) (n = 3) or LNA-anti-miR-192 (LNA) oligonucleotides (n = 4). Scale bar, 20 µm. x400 (10 × 40) magnification. (**C**) Representative immunostaining and quantification for Atg5 or p62 and glomerular area from cortical sections of control mice treated with NC oligonucleotides (Control-NC) (n = 3), STZ-injected diabetic mice treated with NC oligonucleotides (STZ-NC) (n = 3), and STZ-injected diabetic mice treated with LNA-anti-miR-192 oligonucleotides (STZ-LNA) (n = 4). (**D**) Western blot and quantification of LC3 levels (LC3-2/LC3-1) from kidney cortex lysates of Control-NC mice (n = 3), STZ-NC mice (n = 3), and STZ-LNA mice (n = 4) 16 weeks post diabetes induction. Uncropped scans are presented in Supplementary Fig. 1. *P < 0.05; **P < 0.01; ***P < 0.001.

We next examined genes associated with autophagy in streptozotocin (STZ)-diabetic mice (model of type 1 diabetes). We previously showed miR-192 promotes MC hypertrophy and renal fibrosis under diabetic conditions and that injection of a locked nucleic acid modified anti-miR-192 oligonucleotide (LNA-anti-miR-192) in STZ-diabetic mice has a renoprotective effect as depicted by decreases in profibrotic gene expression, renal fibrosis, hypertrophy and proteinurea^42^. Although aberrant autophagy has been implicated in the pathogenesis of DN^29^, the role of miRNAs in regulating autophagy has not been studied intensively. Because miR-192 has been implicated in the pathogenesis of DN, we next wanted to determine whether it also regulates autophagy under diabetic conditions. We therefore examined autophagy factors in kidney cortical lysates from control and STZ-diabetic mice (at 2 and 16 weeks post diabetes induction) that were injected with negative control (NC) or LNA-anti-miR-192 oligonucleotides. Results showed that expression of Atg12 was significantly decreased in STZ-diabetic (STZ-NC) mice compared to control mice injected with NC oligonucleotides (Control-NC) just 2-weeks post diabetes induction (Fig. 2A). This effect was reversed in kidney cortices of STZ-diabetic mice injected with LNA-anti-miR-192 oligonucleotides (STZ-LNA) compared to STZ-NC mice (Fig. 2A). Although there was no significant difference in *Atg7* expression between Control-NC and STZ-NC mice, *Atg7* expression was significantly higher in cortical lysates from STZ-LNA mice compared to STZ-NC mice (Fig. 2A). LC3 expression was also significantly decreased in STZ-NC mice compared to Control-NC mice although it did not increase significantly in STZ-LNA mice at 2 weeks. At 16 weeks post diabetes, *Atg12* expression was again significantly decreased, as also LC3 in STZ-NC mice compared to Control-NC mice and this effect was significantly reversed in kidneys of STZ-LNA mice (Fig. 2B). Although Atg1 and Becn1 expression were not significantly decreased in STZ-NC mice, their expression was significantly increased in cortical lysates of STZ-LNA mice compared to STZ-NC mice (Fig. 2B). Other tested genes were not affected.

Atg5 expression was decreased significantly in glomeruli from STZ-NC mice compared to Control-NC mice, while Atg5 expression was significantly increased in glomeruli from STZ-LNA-anti-miR-192 mice compared to STZ-NC mice (Fig. 2C). Next we performed immunostaining for p62 using cortical sections from these mice. p62 expression was increased significantly in glomeruli from STZ-NC mice compared to Control-NC mice (indicating a decrease in autophagy), whereas p62 expression was significantly decreased in glomeruli from STZ-LNA-anti-miR-192 mice compared to STZ-NC mice indicating a reversal in autophagy repression by miR-192 inhibition (Fig. 2C). Glomerular area was significantly increased in STZ-NC mice compared to Control-NC mice as expected (a key feature of early DN) and this was significantly decreased in STZ-LNA mice compared to STZ-NC mice (Fig. 2C). We also analyzed the conversion of LC3-1 to LC3-2 by western blot using kidney cortical lysates from these mice. We observed a decrease in conversion of LC3-1 to LC3-2 in STZ-NC mice compared to Control-NC mice while this conversion was slightly higher in STZ-LNA mice compared to STZ-NC mice, although this result was not statistically significant due to variability in mice kidney cortex samples (Fig. 2D). These results indicate that the expression of certain autophagy genes is also decreased *in vivo* in the kidneys of a type 1 diabetes (STZ) mouse model and that inhibition of miR-192 *in vivo* can reverse this effect.

### Decreased autophagy gene expression in kidneys of WT STZ-diabetic mice is rescued by genetic knock-out of miR-192

**Figure 3:**
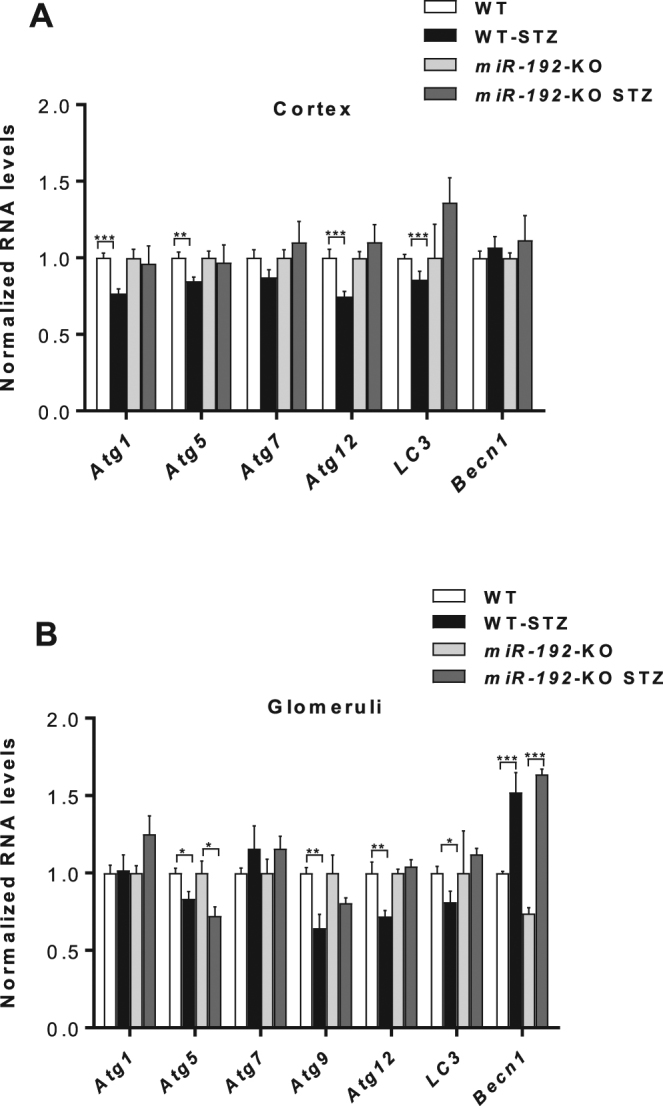
Decreased expression of key autophagy genes in kidneys of WT-STZ mice compared to WT controls is not observed for majority of genes in miR-192-KO-STZ mice compared to miR-192-KO control mice.qRT-PCR analysis of autophagy genes in (**A**) kidney cortex lysates and (**B**) kidney glomerular lysates; from WT control (n = 6) and WT-STZ (n = 5) mice and miR-192-KO control (n = 3) and miR-192-KO-STZ (n = 6) mice 22 weeks after induction of diabetes. *P < 0.05; **P < 0.01; ***P < 0.001.

Since inhibition of miR-192 with LNA-anti-miR-192 oligonucleotides showed a reversal of the reduced expression of certain autophagy genes in a type 1 diabetes mouse model, we next examined whether *miR-192* deletion *in vivo* (using *miR-192* KO mice) can have similar effects on autophagy genes under diabetic conditions. We analyzed expression of autophagy genes from cortical and glomerular lysates from either WT or *miR-192*-KO mice (control and STZ- induced diabetes) at 22 weeks post diabetes induction. *Atg1*, *Atg5*, *Atg12 and LC-3* levels were all significantly decreased in cortical lysates from WT diabetic mice (WT-STZ) compared to non-diabetic control mice (WT) (Fig. 3A). However, expression of these genes was not decreased in cortical lysates from diabetic *miR-192*-KO diabetic mice (*miR-192*-KO STZ) compared to respective non-diabetic controls (miR-192-KO) (Fig. 3A). In glomerular lysates *Atg9*, *Atg12 and LC3* gene expression were significantly decreased in WT-STZ mice but not in *miR-192*-KO STZ mice compared to respective controls. *Atg5* expression was decreased in glomerular lysates from both WT-STZ mice and *miR-192*-KO STZ compared to respective controls. However, *Becn1* expression was significantly increased under diabetic conditions in both WT and *miR-192*-KO mice (Fig. 3B), indicating involvement of other mechanisms for *Becn1* regulation. These results show that certain autophagy genes are decreased in kidneys of diabetic mice also at 22 weeks post diabetes induction and that this decrease is not observed in kidneys of miR-192-KO diabetic mice, indicating again that miR-192 could play a role, at least in part, in regulating the process of autophagy under diabetic conditions.

### Markers of autophagy are decreased in kidneys of WT STZ-diabetic mice but not in kidneys of miR-192-KO STZ-diabetic mice

**Figure 4:**
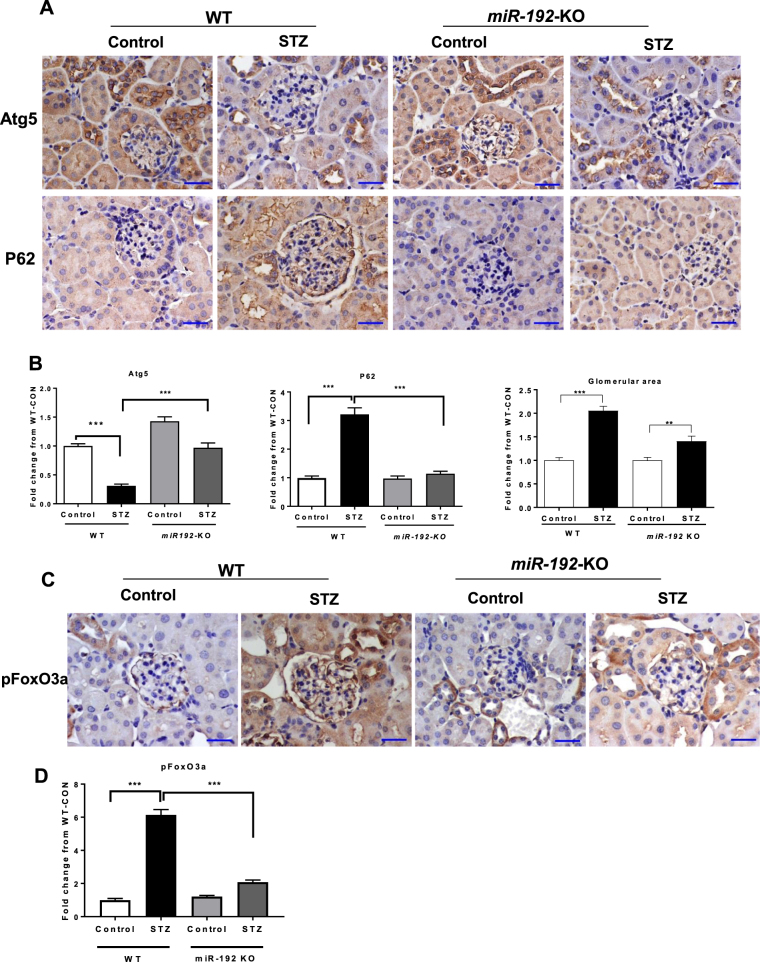
Autophagy is not decreased in kidneys of STZ-injected type-1 diabetic miR-192-KO mice compared to control mice. (**A**) Representative immunostaining and quantification for p62, Atg5 and glomerular area from cortical sections of 22-week old control and STZ-injected -WT and *miR-192-KO* mice; n = 5 per group. Scale bar, 20 µm. x400 (10 × 40) magnification. (**B**) Bar graphs showing quantification for p62, Atg5 and glomerular area. **P < 0.01; ***P < 0.001. (**C**) Representative immunostaining and quantification for pFoxO3a from cortical sections of 22-week old control and STZ-injected -WT and *miR-192-KO* mice; n = 5 per group. Scale bar, 20 µm. (**D**) Bar graphs showing quantification for pFoxO3a. ***P < 0.001.

Next we examined the protein expression of well-known markers of autophagy in WT and *miR-192*-KO, control and STZ-diabetic mice by performing immunostaining for Atg5 and p62 using kidney cortical sections from these mice. Expression of Atg5, an important protein regulating the autophagy pathway, was decreased in glomeruli of WT STZ-diabetic mice compared to WT-control mice. However this decrease in Atg5 expression was not observed in *miR-192*-KO STZ-diabetic relative to *miR-192*-KO controls (Fig. 4A,B). p62 expression was significantly increased in glomeruli of WT STZ-diabetic mice compared to WT-control mice indicating a decrease in autophagy under diabetic conditions. However, this increase in p62 expression was not seen in glomeruli from *miR-192*-KO STZ-diabetic mice compared to respective control mice (Fig. 4A,B**)**. These results indicate that autophagy is decreased in glomeruli under diabetic conditions and this is reversed by deletion of *miR-192*. Furthermore, the decrease in autophagy correlated with the increase in glomerular area in WT STZ-diabetic mice compared to controls. Increase in glomerular area was much lower in *miR-192*-KO STZ-diabetic mice compared to respective controls (Fig. 4B).

### Phosphorylated FoxO3a (pFoxO3a) is increased in kidneys of WT STZ-diabetic mice but not in kidneys of miR-192-KO STZ-diabetic mice

FoxO3a is a direct upstream transcription factor that regulates several Atg genes through Fork-head response elements in their promoters^43,44^. As FoxO3a can be phosphorylated and inactived by Akt kinase, phosphorylated FoxO3a (pFoxO3a) was examined by immunostaining (Fig. 4C). pFoxO3a levels were significantly increased in renal sections including glomeruli of WT STZ-diabetic mice compared to WT-control mice. However, this increase in pFoxO3a was significantly attenuated in glomeruli from *miR-192*-KO STZ-diabetic mice compared to respective control mice (Fig. 4C,D). These results verify that FoxO3a is a critical downstream autophagy regulating factor controlled by miR-192-mediated signal cascade in the diabetic kidney.

### Autophagy gene expression is decreased in MMC treated with TGF-β and certain genes are decreased with miR-192 and miR-217 mimic oligonucleotides

**Figure 5:**
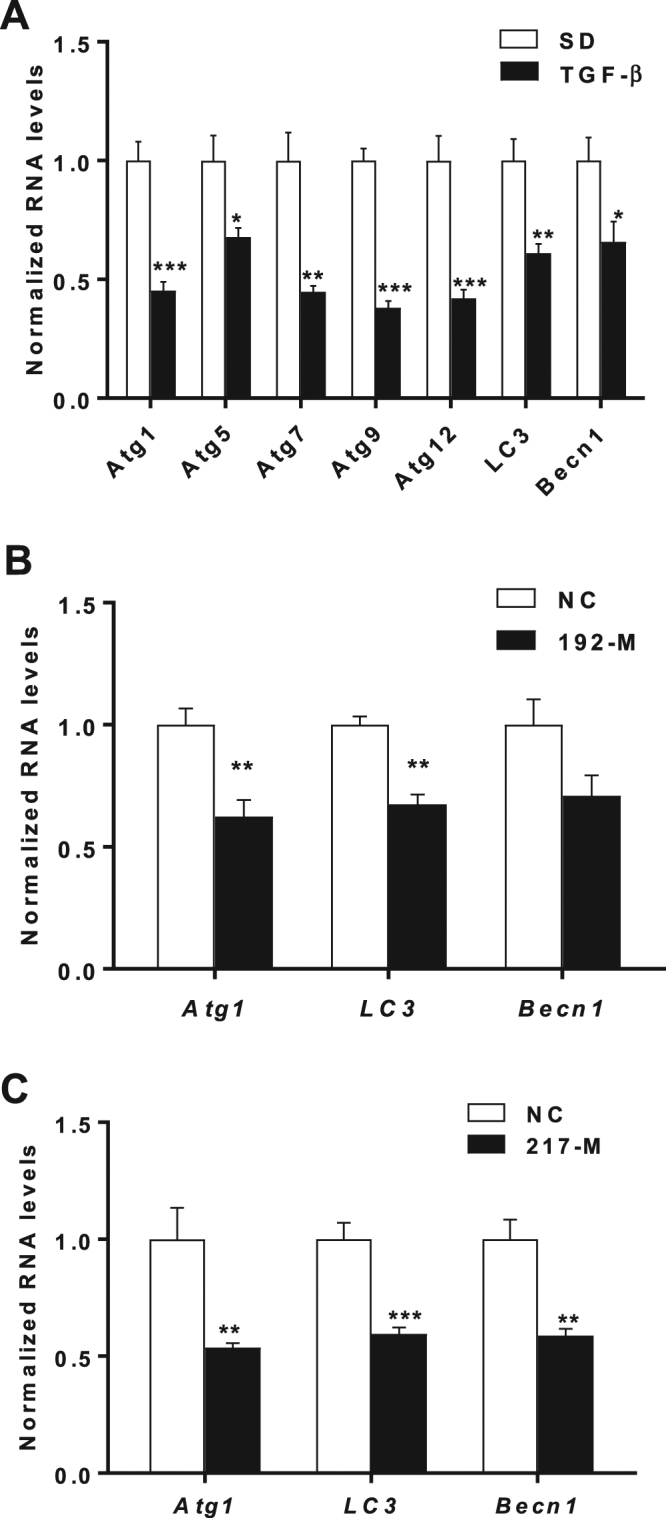
Autophagy gene expression in MMCs treated with TGF-β or with miR-192-M and miR-217M.qRT-PCR analysis of autophagy genes in (**A**) WT-MMCs treated with TGF-β (n = 3) (**B**) WT-MMCs transfected with either negative control (NC) or miR-192 mimic oligonucleotides (192-M) (n = 3). (**C**) WT-MMC transfected with either negative control (NC) or miR-217 mimic oligonucleotides (217-M) (n = 3). Error bars SEM *P < 0.05; **P < 0.01; ***P < 0.001.

Since miR-192 plays a role in the pathogenesis of DN downstream of TGF- β, we wanted to test whether TGF-β regulates autophagy gene expression via miR-192 in MMCs. TGF-β treatment of MMCs led to a significant decrease in the expression of multiple autophagy genes compared to controls (Fig. 5A). Next, in order to test whether miR-192 directly regulates expression of some of these genes, we transfected MMCs with miR-192 mimic oligoneucleotides (192-M) or negative control oligonucleotides (NC) and found that miR-192 significantly decreased the expression of *Atg1 and LC3* in these cells (Fig. 5B). We previously reported that miR-217 is another miRNA induced by TGF-ß, miR-192 and diabetic conditions that are involved in the signaling cascade downstream of TGF-ß and miR-192 in MMCs and also regulates the pathogenesis of DN^40^. We transfected MMCs with either NC or 217-M and observed that expression of *Atg1*, *LC3 and Becn1* were significantly decreased by miR-217-M compared to NC (Fig. 5C). Overall, these results indicate that autophagy gene expression is regulated by the TGF-β pathway in MMCs and one of the mechanisms involved could be through downstream miRNAs, namely miR-192 and miR-217.

### GFP-RFP-LC3 puncta are decreased in WT-MMCs compared to miR-192-KO MMCs following TGF-β treatment

**Figure 6:**
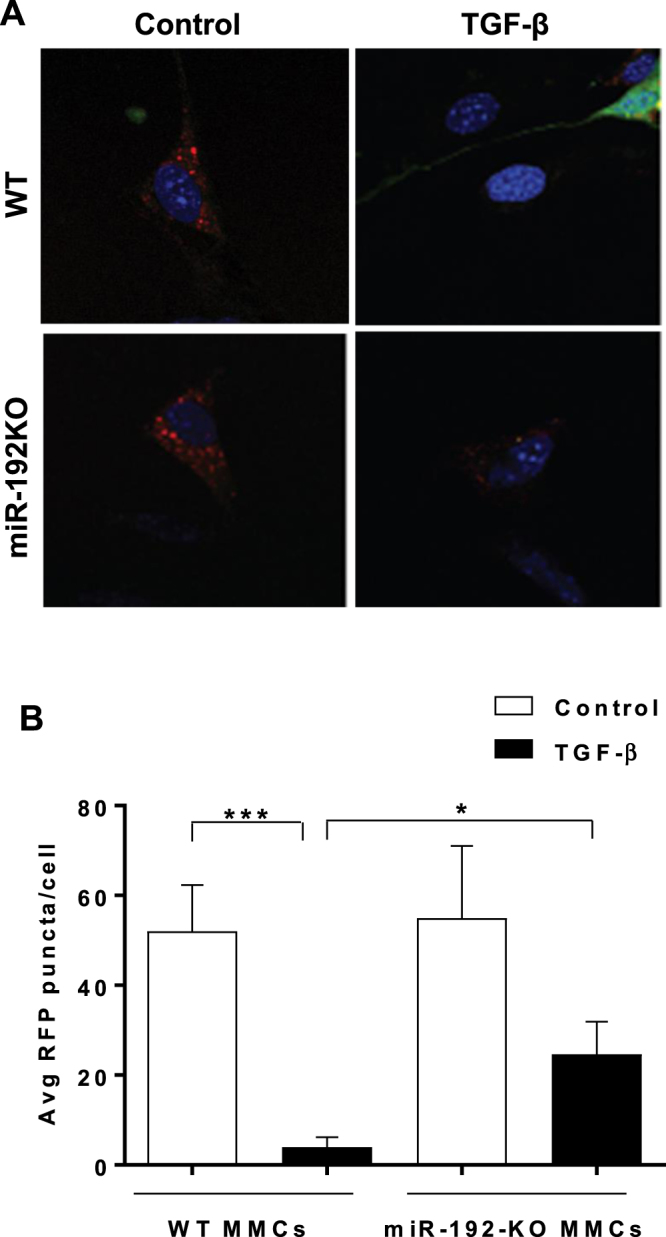
Autophagosome formation is inhibited to a greater extent in WT-MMCs compared to miR-192-KO MMCs following TGF-β treatment. (**A**) Representative confocal microscopy images showing RFP-LC3 puncta in WT or miR-192-KO MMCs transfected with GFP-RFP-LC3 plasmid (1 µg) followed by TGF-β treatment of these cells for 24 hrs. x1000 (10 × 100 oil) magnification. (**B**) Bar graph showing quantification of RFP-LC3 puncta in WT and miR-192-KO MMCs. *P < 0.05; ***P < 0.001.

Next we analyzed the effect of TGF-β treatment on the formation of GFP-RFP-LC3 puncta in MMCs derived from WT mice and *miR-192*-KO mice. We observed that the number of RFP-LC3 puncta, indicative of autolysosomes^10^ were decreased following TGF-β treatment in both WT as well as *miR-192*-KO MMCs. However, the decrease in these RFP-LC3 puncta was significantly lesser in TGF-β treated *miR-192*-KO MMCs as compared to TGF-β treated WT MMCs (Fig. 6A,B). The number of RFP-LC3 puncta were counted and the average number of RFP puncta per cells were plotted as a bar graph (Fig. 6B). These results indicate that autophagy in MMC is reduced by TGF-ß and also by its downstream effector miR-192 in MMCs under diabetic conditions.

## Discussion

In this study, we show for the first time that TGF-β and miRNA-192 regulate autophagy gene expression *in vitro* in MMCs as well as *in vivo* in kidneys of diabetic mice. Autophagy gene expression as well as certain markers of autophagy was decreased in kidneys of STZ-diabetic mice and *db/db* mice compared to controls. The effect of diabetes on autophagy gene expression was ameliorated in kidneys of STZ-diabetic miR-192-KO mice as well as in kidneys of WT STZ-diabetic mice treated with LNA-anti-miR-192 oligonucleotides, compared to respective controls (Figs 2–4).

We observed that several autophagy genes which were decreased under diabetic conditions in WT mice (compared to WT controls) were not decreased in kidneys of *miR-192*-KO STZ-diabetic mice (compared to *miR-192*-KO controls) namely, *Atg1*, *Atg5*, *Atg12 and LC3*, at 2 weeks post diabetes, and *Atg9*, *Atg12 and LC3* at 22 weeks post diabetes (Fig. 3). These results suggest that miR-192 is involved, at least in part, in the regulation of expression of these genes under diabetic conditions. However, at 22 weeks post diabetes, glomerular *Atg5* and *Becn1* were regulated differently indicating another mode of regulation for these genes. Since *Atg5* gene expression decreased in *miR-192*-KO STZ-diabetic glomeruli but immunostaining did not show a parallel decrease in Atg5 protein expression in these mice, some other unknown post-transcriptional regulation may be involved in the Atg5 regulation (Figs 3 and 4). Responses of *Becn1* were different between LNA-anti-miR-192 and miR-192KO mice (Figs 2 and 3) suggesting that the regulation of Becn1 by miR-192 inhibition with a miR-192 inhibitor may be different from that in mice in which miR-192 expression is genetically reduced from the birth. These results also suggest that Becn1 may not be a major effecter of autophagy-related hypertrophy in miR-192 mediated signal cascade in these mouse models of DN.

p62 protein can bind to LC3 and is selectively degraded during autophagy. In addition, ubiquitinated proteins can bind to p62 and are recruited to autophagosome membranes. Hence p62 serves as a connection between LC3 and ubiquitinated substrates and can be used as a monitor of autophagic flux^27,45^. Accumulation of p62 is indicative of a defect in the autophagic pathway. Our immunostaining results showed an accumulation of p62 in glomeruli of *db/db* and WT STZ-diabetic, indicating that diabetic conditions cause a decrease in glomerular autophagy, likely in mesangial compartments. Defects in the autophagy pathway have been implicated in the pathogenesis of type-2 diabetes^46,47^. Interestingly, upon inhibition of miR-192 *in vivo* using LNA-anti-miR-192 oligonucleotides, as well as in *miR-192*-KO mice, p62 accumulation was significantly decreased in glomeruli under diabetic conditions compared to control mice indicating a reversal of autophagic suppression in these mice. Inhibition of miR-192 may enhance autophagy in MMC under diabetic conditions.

Autophagy is a process by which there is bulk protein degradation and recycling of long-lived proteins and damaged organelles^7–9,11,16,17^. A decrease in autophagy leads to accumulation of damaged proteins and organelles disturbing cellular homeostasis and is known to play a role in the pathogenesis of diabetes, liver injury, myopathy and neurodegeneration^45^. Previous studies have identified miRNAs which regulate cardiac cell autophagy and subsequently hypertrophy, as therapeutic targets^48^. Notably as observed in our previous study^38^ as well as in the current study, increases in glomerular hypertrophy (as measured by glomerular area) under diabetic conditions was attenuated in kidneys of diabetic mice in which miR-192 was inhibited (with anti-miRNAs) or genetically knocked out (miR-192-KO mice). We have previously shown that inhibition or knock down of miR-192 plays a protective role in DN by lowering kidney fibrosis and proteinuria^38,42^. Endoplasmic reticulum stress induces autophagy in renal proximal tubular cells^12^. We reported that chemically-modified antisense oligonucleotides (GapmeRs) targeting a long noncoding RNA, lncMGC, which is the host RNA of the miR-379 cluster, ameliorated fibrosis and hypertrophy in early stage of DN through inhibition of ER stress^13^. Since autophagy regulates cellular hypertrophy, results from the current study indicate that autophagy may be one of the pathways that plays a protective role in this mechanism.

We have previously shown that miRNA circuits downstream of TGF-ß, which include miR-192 and miR-217, induce MC hypertrophy as a consequence of Akt activation and downregulation of PTEN^40^. We also demonstrated another mechanism of Akt activation through downregulation of FOG2 by miR-200b/c downstream of TGF-β in MMCs^39,49^. The Akt inhibitor (MK-2206) ameliorated kidney fibrosis and hypertrophy in glomerular mesangial cells through inhibition of p300 phosphorylation and histone/Ets1 acetylation^50^. In the current study we used MMCs derived from WT mice and *miR-192*-KO mice to analyze the effect of TGF-β on autophagy genes *in vitro*. Our current results indicate that TGF-β down-regulates several autophagy genes in MMCs. Furthermore, some of these genes are down-regulated by oligonucleotide mimics of miRNAs downstream of TGF-β namely miR-192 and miR-217. Several mechanisms could regulate the process of autophagy under these conditions and detailed molecular analysis is required in order to identify these pathways. Proteins downstream of Akt such as mTOR and GSK3B are regulators of hypertrophy and autophagy^7–9,11,16,17^.

**Figure 7:**
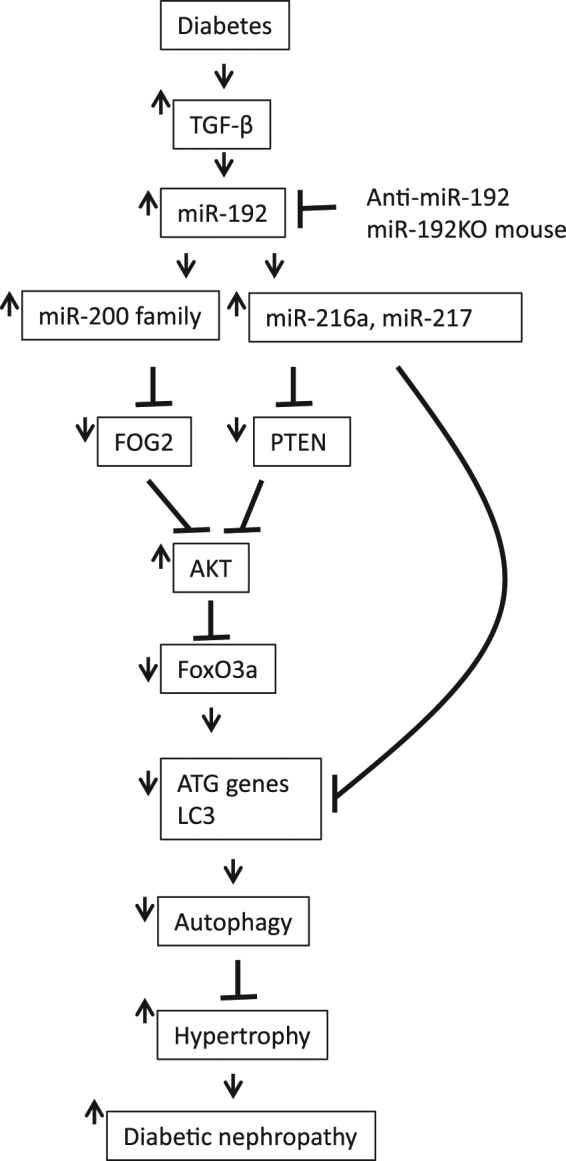
Proposed model showing the regulation of autophagy by miR-192-mediated cascade under diabetic conditions leading to the pathogenesis of DN. Please read main text for details.

Furthermore, Akt phosphorylates and inhibits FoxO3a, a transcription factor known to increase transcription of autophagy genes in hematopoietic stem cells, and Akt kinase promotes cell survival and hypertrophy^4,40,51^. Activation of FoxO3a in atrophying muscles has also been shown to induce autophagy gene expression^43,44^. Previously, we reported that pFoxO3a levels are significantly increased in glomeruli from STZ-diabetic mice and rats, suggesting decreased FoxO3a transcriptional activity in diabetes^4,40^. In the current study, we again found that pFoxO3a levels were significantly increased in glomeruli of WT STZ-diabetic mice. However, notably, this increase in pFoxO3a was significantly inhibited in glomeruli from *miR-192*-KO STZ-diabetic mice (Fig. 4C,D). These results provide further experimental evidence supporting FoxO3a as a critical downstream factor (also related to autophagy) in the miR-192-mediated signaling cascade. Hence inhibition of miR-192 and subsequent decreased Akt activity and activation of downstream regulators of autophagy could be one of the mechanisms by which autophagy is increased following treatment of diabetic mice with LNA-anti-miR-192 oligonucleotides or in miR-192-KO mice (Fig. 7).

Moreover, increased Akt activity leads to an increase in mTORC1 activity which in turn inhibits autophagy. Loss of miR-192 and hence decreased Akt activation with subsequent reduction in mTOR activity could also contribute to reversal of autophagy suppression observed in the diabetic *miR-192*-KO mice^15,40^. miR-217 targets the 3′UTR of FoxO3A in endothelial cells^52^. Interestingly, another miRNA, miR-216a, that is also downstream of TGF-β signaling and miR-192, has been shown to target BECN1 and ATG5 expression in endothelial cells and Becn1 in pancreatic cancer cells^40,53,54^. miR-216a also plays a pathogenic role in DN by up-regulating collagen genes through TFE3 by targeting YBX-1^55^. Interestingly, TFEB and TFE3 are involved in the process of ER stress and autophagy^56,57^. Thus, it would be interesting to further study whether these miRNAs regulate autophagy in MMCs under diabetic conditions. Overall, it is likely that the inhibitory effect of miR-192 on autophagy genes is mediated by these miRNAs (miR-216a, 217 and 200) which are induced by miR-192 (Fig. 7)^39,40^.

We have previously shown that *miR-192*-KO mice are protected from the pathogenesis of DN compared to WT mice^38^. In the current study our results demonstrate that downregulation of autophagy by miR-192 and subsequent increase of glomerular hypertrophy could be one mechanism that leads to complications associated with DN. We propose a model of autophagy regulation in MMCs under diabetic conditions in which upregulation of TGF-β and miR-192 cascade and subsequently increased Akt activation could lead to inhibition of autophagy mediated either directly by miR-192, miR-217 and other downstream miRNAs targeting some of the autophagy genes, or via transcriptional regulation of these genes due to phosphorylation and inhibition of FoxO3a activity by Akt activation (Fig. 7).

Taken together, these results indicate that miR-192 decreases autophagy in MMC under both type-1 and type-2 diabetic conditions and that inhibition of miR-192 with LNA-anti-miR-192 oligonucleotides or deletion of miR-192 can lead to reversal of autophagic suppression along with decreased glomerular hypertrophy (Fig. 7). Hence inhibition of miR-192 leading to the activation of autophagy and reduction in glomerular mesangial hypertrophy in early stages of DN could be a useful therapeutic strategy.

## Materials and Methods

### Animal studies

All animal studies were performed according to approved Institutional Animal Care and Use Committee (IACUC) protocols. Type-2 diabetic *db/db* mice and genetic control *db/*+ mice (10–12 week old) were from Jackson Laboratories. C57BL/6 mice were made diabetic by once daily injections of 50 mg/kg of streptozotocin (STZ) for five subsequent days as described previously^42^. Mice injected with control citrate buffer vehicle were used as controls. Wild type and *miR-192*-KO mice were obtained as described previously^38^. Renal cortical sections from *db*+ and *db/db* mice, 16 week STZ-injected diabetic mice injected with LNA-anti-miR-192 or control LNA-anti-miR-239b oligonucleotides, and from wild type and *miR-192*-KO mice were obtained as described previously^38,42^.

### Cell culture and materials

Primary mouse MC (MMCs) were isolated and cultured using renal glomeruli from wild type mice and *miR-192*-KO mice as described before^13,38^. Recombinant human TGF-β1 was from R&D Systems, Inc. (Minneapolis, MN, USA). miR-192 mimics (192-M), miR-217 mimics (217-M), negative control oligonucleotides (NC) were obtained from Dharmacon (Lafayette, CO). RFP-GFP-LC3 plasmid was obtained from Addgene. MC were transfected with miRNA mimics or RFP-GFP-LC3 plasmid using a Nucleofector and Basic Nucleofector Kit (Lonza). Cells were serum depleted and treated with TGF-β for the indicated time periods.

### Quantitative RT-PCR (q-PCR)

Real time q-PCR analysis was performed using a 7300 or 7500 Realtime PCR System (Applied Biosciences, Foster City, CA, USA) with SYBR green PCR master mix and three-step standard cycling conditions. Briefly, 0.2 ug to 0.5 µg of total RNA was used to synthesize cDNA using a GeneAmp PCR kit from Applied Biosystems. 18sII primers (Ambion - Thermo Fisher Scientific) or Cyclophilin A were used as internal control and quantitative analysis was performed using the ∆∆CT method^13,58^. The following sequence specific mouse PCR primers were used: Atg1 forward 5′-CAAGCTGTGCATTGAGAGGA-3′; reverse 5′-AGGACAGTCTGCCAGGTCTC-3′; Atg5 forward 5′-CATCAACCGGAAACTCATGG-3′; reverse 5′-CGGAACAGCTTCTGGATGAA-3′; Atg7 forward 5′-GCCCTGCCCTACTTCTTATTC-3′; reverse 5′-AGGGATCGTACACACCAACTG-3′; Atg9 forward 5′-CGAGGCTGGTAACTGGAATCT-3′; reverse 5′-GCTCCATGGACAGTTTCTGC-3′; Atg12 forward 5′-CCCGGAGACACCAAGAAAA-3′; reverse 5′-GTCAATGAGTCCTTGGATGGTC-3′; LC3 forward 5′-GCCTGTCCTGGATAAGACCA-3′; reverse 5′-GGTTGACCAGCAGGAAGAAG-3′; Becn1 forward 5′-CAGCTGGACACTCAGCTCAA-3′; reverse 5′-CTCTGCAGCTGCTCACTGTC-3′; Cyclophilin A (CypA) forward 5′-GACCAAACACAAACGGTTCC-3′; reverse 5′-CTCCATGGCTTCCACAATG-3′.

### Western Blot Analysis

Protein extraction and subsequent analysis was performed as described before^38^. Lysis buffer (20 mM TrisHCl (pH 7.4), 150 mM NaCl,1% Triton) was used to make lysates for LC3 western blot analysis. Following antibodies were used at 1000 × dilution: LC3 (Novus Biological, Littleton CO) and α-Tubulin (Santa Cruz Biotechnology, Santa Cruz, CA).

## Immunohistochemistry

To determine the expression of Atg5 and p62 in the glomerulus, immunostaining of paraffin-embedded renal cortical sections was performed as described previously^13,42^. Antibodies used were for Atg5 (Abcam, ab108327, 1:150), p62 (Abcam, ab155686, 1:100) and pFoxO3a (Cell Signaling, #9466, 1:100). Images were captured using an Olympus DP72 Microscope Digital Camera and processed with ImageJ (fiji-win32) software. Glomerular area and mesangial expansion index were quantified using Image-Pro Plus 5.1 software (Media Cybernectis, In, Bethseda, MD).

### Determination of autophagosome formation

MMCs were transfected with 1 µg RFP-GFP-LC3 plasmid using a Nucleofector and Basic Nucleofector Kit (Lonza). Cells were plated in 12 well plates with cover-slips and serum depleted for a total of 48hrs followed by a 24 hr TGF-β treatment. Cover-slips were transferred and fixed onto glass slides and formation of RFP-LC3 puncta was observed using a confocal microscope (Zeiss). Average number of RFP-LC3 puncta were counted per cell (n = 10–15 cells per condition).

### Cell number and cellular protein levels

MC were trypsinized and counted using a Coulter Counter with 100-µm aperture (Beckman). Cells were lysed and total protein content was measured using the Bio-Rad Protein Assay (Bio-Rad).

### Statistical analyses

PRISM Software (Graphpad, San Diego, CA) was used for data analysis. Results are expressed as mean ± s.e.m from multiple experiments. Student’s t-tests were used to compare two groups. Statistical significance was detected at the 0.05 level. Based on our previously published experience^4,13,38,40,42,49,58^, sample size was determined to have enough power (at least 80%) to detect an estimated difference between two groups. Our actual results with the sample size used showed statistical significance.

## Electronic supplementary material

Supplementary Fig1
