# Supplementary material for: Reduced Autophagy by a microRNA-mediated Signaling Cascade in Diabetes-induced Renal Glomerular Hypertrophy
Source: Sci Rep. 2018 May 3;8:6954. doi: 10.1038/s41598-018-25295-x (PMC5934412; doi:10.1038/s41598-018-25295-x)
Supplement: Supplementary file 1 — Supplementary Fig1 [file 41598_2018_25295_MOESM1_ESM.pdf]

Reduced Autophagy by a microRNA-mediated Signaling Cascade in Diabetes-induced Renal Glomerular Hypertrophy

Supriya Deshpande, Maryam Abdollahi, Mei Wang, Linda Lanting, Mitsuo Kato and Rama Natarajan

Department of Diabetes Complications and Metabolism, Diabetes Metabolism Research Institute and Beckman Research Institute of City of Hope, Duarte, California 91010, USA

Correspondence to:

Rama Natarajan, rnatarajan@coh.org or

Mitsuo Kato, mkato@coh.org

Department of Diabetes Complications and Metabolism

Diabetes & Metabolism Research Institute

Beckman Research Institute of the City of Hope

1500 East Duarte Road,

Duarte, CA 91010

Tel: 626-218-2289, 626-218-3996

Fax: 626-301-8136

Supplementary Figure 1

Fig.1E

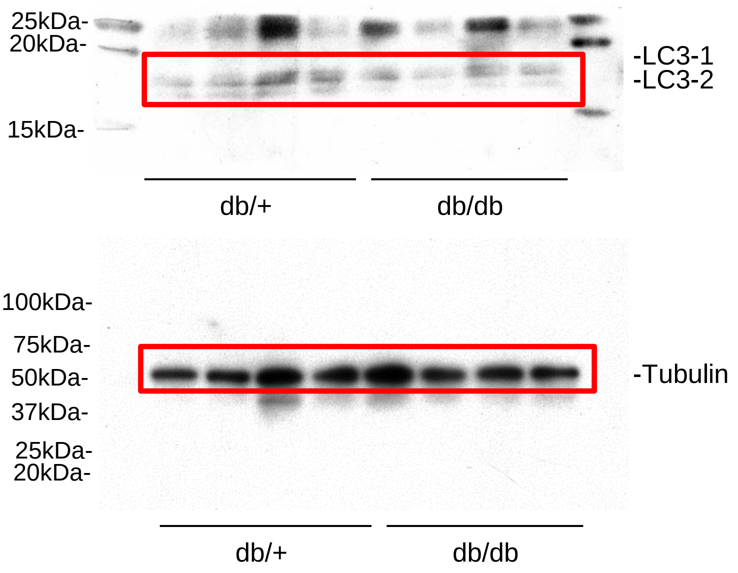

Fig.2D

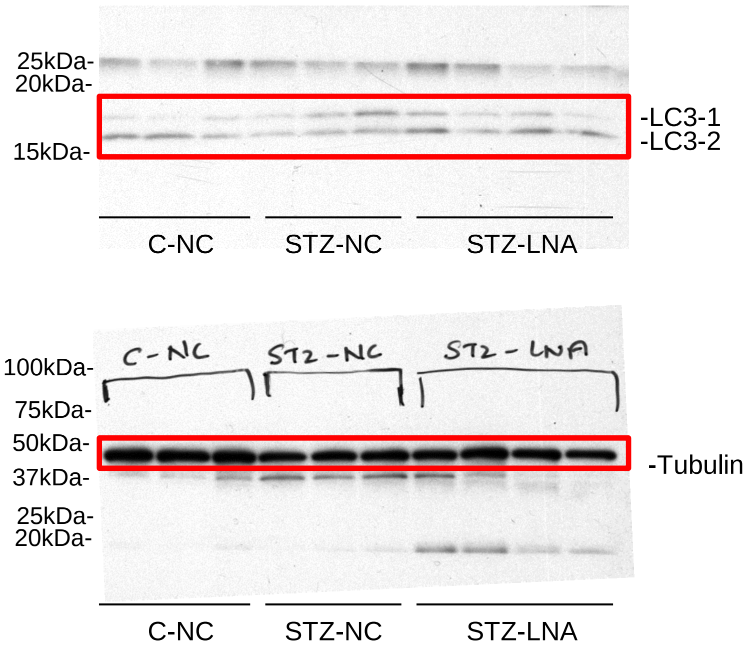

Supplementary Figure 1. Wider (uncropped) scans of blots (Fig.1E & Fig.3D).
